# A multi-center cross-sectional study of Chinese Herbal Medicine-Drug adverse reactions using active surveillance in Singapore’s Traditional Chinese Medicine clinics

**DOI:** 10.1186/s13020-024-00915-z

**Published:** 2024-03-07

**Authors:** Chester Yan Jie Ng, Yan Zhao, Ning Wang, Kwan Leung Chia, Chun Huat Teo, William Peh, Pansy Yeo, Linda L. D. Zhong

**Affiliations:** 1 School of Biological Sciences, Nanyang Technological University, 60 Nanyang Drive, Singapore, 637551 Singapore; 2 School of Chinese Medicine, Li Ka Shing Faculty of Medicine, The University of Hong Kong, Hong Kong, Hong Kong; 3 Woodcroft Medical Centre, 1 Sir James Hardy Way, Woodcroft, SA 5162 Australia; 4 Singapore Thong Chai Medical Institution, 50 Chin Swee Road #01-01, Singapore, 169874 Singapore; 5 Operation and Medical Department, Singapore Chung Hwa Medical Institution, 640 Lorong 4 Toa Payoh, Singapore, 319522 Singapore; 6 Chong Hoe Healthcare, 144 Upper Bukit Timah Rd, #02-14, Singapore, 588177* Singapore

**Keywords:** Adverse Events, Traditional Chinese Medicine, Active Surveillance, Epidemiology, Drugs

## Abstract

**Background:**

This study aimed to investigate the rates and causality of patient-reported adverse events (AEs) associated with concomitant Chinese Herbal Medicine (CHM) and Western Medicine prescription drug (WMPD) consumption through active surveillance in Singapore’s Traditional Chinese Medicine (TCM) clinics.

**Methods:**

A cross-sectional study was conducted at five TCM clinics across Singapore from 8th May till 8th July 2023. Patients were screened to determine rates of CHM and WMPD consumption, and then interviewed if an AE was reported. An expert committee assessed the AE reports to determine causality. Along with descriptive statistics, odds ratios were calculated to determine AE occurrence likelihoods for patients who consumed both CHM and WMPD compared to CHM consumption alone.

**Results:**

1028 patients were screened and 62.65% of them reported concurrent CHM-WMPD consumption. Patients who consumed CHM and WMPD were 3.65 times more likely to experience an AE as compared to CHM consumption alone. 18 AE reports were adjudicated, with most AEs deemed unlikely due to CHM consumption.

**Conclusions:**

A large proportion of patients consumed CHM and WMPD concurrently, thus increasing their risk of experiencing AEs compared to those consuming CHM only. Active surveillance is applicable for detecting AEs, collecting data for causality assessment, and analysis.

## Introduction

### Background

With increasing popularity and usage of Traditional Chinese Medicine (TCM) worldwide, the consumption of Chinese Herbal Medicine (CHM) has also increased [1, 2]. Many developed countries have shown interest in complementary and alternative medicine (CAM), thus generating an increase in demand for complementary medical therapies [3]. In Singapore, although Western Medicine (WM) is the main mode of healthcare, TCM also enjoys considerable popularity as a complementary therapy. The Singapore National Health Survey 2010 revealed that 39.6% of the population respondents had visited a TCM physician, 26.0% sought treatment for general well-being, 25.8% for acute minor injuries such as sprains, 20.6% for chronic aches and pain like headaches, back pain and rheumatism, and 17.5% for acute minor illnesses [4]. TCM is also the most widely used form of CAM in Singapore, accounting for 88% of total CAM use [5]. One probable explanation for this high rate of use is TCM's long history, and a general belief that TCM is a safe treatment alternative for preventative care and chronic disease management [6]. Furthermore, the Ministry of Health (MOH) estimated that about 45% of the population had consulted a TCM practitioner in the past in 1994, and 7 years later, it was revealed that the rate had increased to 67%. Hence, TCM use is progressively growing amongst the Singapore population [7].

### Increased risks of herb-drug interactions

Increasing CHM consumption necessitates a greater awareness of potential risks that may arise from the concurrent use of CHM and WM prescription drugs (WMPD). Any negative or undesired sign, symptom, or disease correlated with the use of a pharmaceutical product, regardless of whether it is related to the product itself, is defined as an adverse event (AE) emerging from consumption. An AE that is suspected to be due to the consumption of a pharmaceutical product is defined as an adverse reaction (AR) [8]. With increasing prevalence of concurrent consumption of CHM with WMPD, patients are at a higher risk of unintended herb-drug interactions, especially with drugs with narrow therapeutic indices such as warfarin [9]. An increase in the frequency of herb-drug interactions could lead to increased occurrences of AEs and ARs, thus impacting patient safety [10, 11]. Therefore, it is crucial to have a system in place to monitor the occurrences of such AE reports, and for the necessary authorities to act and assess the underlying reasons behind these AEs to minimize repeat events.

### Current methods to detect AEs and ARs

Currently, regulatory agencies rely primarily on passive surveillance systems to detect the presence of AEs and ARs, a common example being surveillance systems to monitor potential adverse effects of vaccines [12, 13]. This model has also been used for other healthcare-related purposes, such as monitoring the safety of point-of-care products, new healthcare products and dengue prevention [14–16]. However, the success of these systems is largely dependent on spontaneous reporting by both patients and healthcare professionals, the individual's discretion in recognizing when an AE should be recorded, and their ability to submit a thorough report for assessment [17, 18]. Other variables influencing the detection of AEs include healthcare professionals not actively inquiring about the patient's AE and medication consumption history, patients’ unwillingness to disclose AEs, and an overall lack of awareness about the risks associated with medicinal product consumption [8, 19–21]. Hence, there is a growing need for an alternative system to complement passive surveillance to actively identify AEs and enhance patient safety [22].

### Active surveillance as an alternative model

In terms of epidemiological surveillance, active and passive surveillance are two main methods of surveillance. In passive surveillance systems, medical professionals in the community and at health facilities report cases to the public health agency, which conducts data management and analysis once the data are received [23]. On the other hand, active surveillance necessitates that public health personnel participate actively in the system and take action to obtain case reports. This may involve calling or visiting health facilities to encourage follow-up or having staff review medical records to identify cases meeting prescribed case definitions. Recent studies have shown that active surveillance systems are effective in collecting AE and AR data [22]. For instance, pharmacy Study Of Natural health product Adverse Reactions (SONAR) was a multicenter population-based observational study in which the researchers partnered with Health Canada, community pharmacists, and pharmacies to implement an active surveillance screening system to detect patient-reported AEs associated with natural health product consumption [24]. The pilot study conducted in Ontario in 2012 discovered that the deployment of active surveillance detected a 3000-fold greater rate of ARs than the passive monitoring approach used by Health Canada during the same time period [22]. Following the pilot study, the subsequent multi-center cross-sectional study conducted in Alberta, and British Columbia (Western Canada) also confirmed that active surveillance had significantly increased AE reporting rates [24]. During the study, community pharmacists screened consecutive patients, or agents of patients who were dropping or picking up prescription medications. Thereafter, patient interviews were conducted for patients who reported and AE to collect meaningful information for full causality assessment of an AE. Laboratory analysis was conducted to support this assessment. Both studies were well received, and feedback from the participating clinics and patients was also positive. Considering the success of this active surveillance model in Canada, we chose to adopt and adapt this model of active surveillance for data collection at local TCM clinics. For this study, we decided to engage TCM physicians and clinic staff to conduct screening of patients. Thereafter, patient interviews were conducted for patients who reported and AE to collect meaningful information before causality assessment of an AE was conducted.

### Significance and objectives

In Singapore, related studies have relied on data-mining techniques to evaluate AEs reported from consumption of CAM products and supplements [25, 26]. These studies were able to reveal trends associated with AE occurrences, and one common consensus was the importance of AE reporting even if causality could not be proven, as the presence of significant clusters of AE reports could compel relevant authorities to take immediate corrective action and investigate the causes of these AEs. However, the effectiveness and feasibility of different methods of AE surveillance has not yet been investigated. This study would also be the first and largest multi-center observational study on CHM-WMPD adverse reaction conducted in Singapore. Through active surveillance, we aimed to determine the proportions of patients using both CHM and WMPD concurrently, and their respective AE rates. Causality assessment would also be conducted on the reported AEs to better understand the underlying reasons behind AEs reported by patients who consume CHM and WMPD.

## Methods

**Fig. 1:**
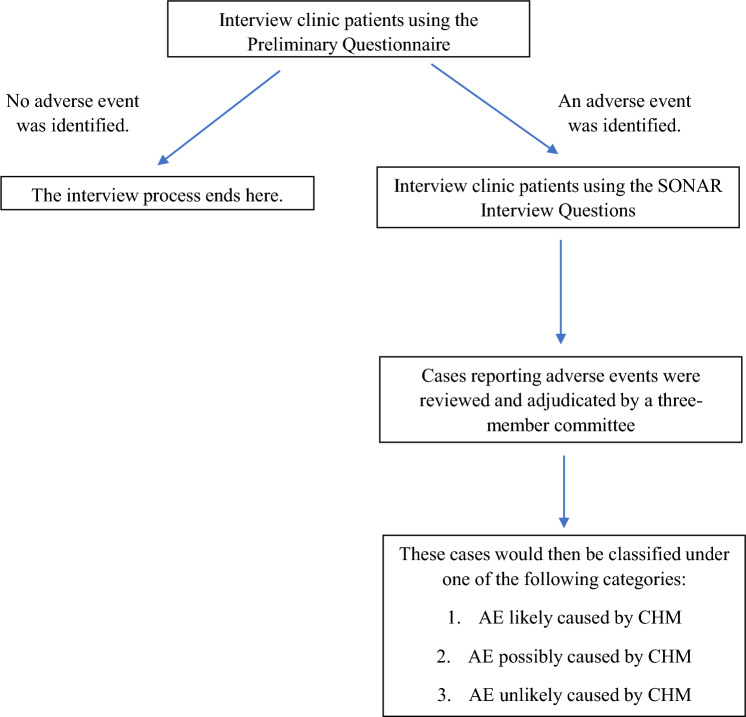
Flowchart depicting the study flow

Firstly, this study protocol was approved by the Institutional Review Board of Nanyang Technological University (Reference Number: IRB-2023-312). The study was then conducted using a two-phase cross-sectional model, and was reported in accordance with the STROBE statement [27, 28]. Phase 1 involved the implementation of an active surveillance model in local TCM clinics and data collection via patient interviews. Thereafter, Phase 2 involved causality assessment of the AEs by a team of medical and pharmacology experts. The study flow is shown in Fig. 1 below.

### Phase 1

Five local TCM clinics were approached to take part in the study for 9 weeks, lasting from 8th May 2023 to 8th July 2023. The clinics involved were: (1) NTU Chinese Medicine Clinic (NTU TCM), (2) Singapore Thong Chai Medical Institution (STCMI), (3) Singapore Chung Hwa Medical Institution (SCHMI), (4) Chong Hoe Healthcare Beauty World Centre Branch (CHHC BWC), and (5) Chong Hoe Healthcare Clarke Quay Central Branch (CHHC CQC). We chose to include commercial (CHHC BWC and CHHC CQC), nonprofit (STCMI and SCHMI), and educational institutions (NTU TCM) to provide a more unbiased sampling of the nation’s TCM patient population. Subsequently, training was provided to the designated staff in charge of data collection, and relevant study materials such as consent forms and patient information packages were provided. Clinic staff were also provided with follow-up and assistance via remote support such as telephone calls and online meetings if necessary.

All adult patients (≥ 21 years of age) who have consumed CHM at the participating clinics were included in the study. Prior to the study, the clinic managers and clinic staff involved in this study were passed copies of the consent form to inform them of the details of the questions to be asked, and they were asked to sign a participation consent form as well. For this study, the primary investigator oversaw and conducted patient recruitment. Research assistants were stationed at the patient waiting area and interviewed consecutive patients to ask if they would be willing to take part in this study. In addition, the physicians involved in this study also assisted with patient recruitment by asking the patients during their consult period. All participation in this study was voluntary and no remuneration was given to the participants. Thereafter, if they agreed to participate in the study, they would be tasked to sign a patient consent form. The patient information package passed to the patients was an information sheet detailing our study details and the aim of our study. It was displayed both in the clinic and passed out to the patients who participated in our study. Patients who could not communicate in either English or Chinese were not included in this study.

During the study, patient confidentiality was ensured as no patient identifiers were collected. All results collected were only by name, and no other private details (such as phone number, email, NRIC, and other ID information) were collected. In addition, the results collected were stored on a password-protected Excel document that was only accessible to the PI.

**Fig. 2:**
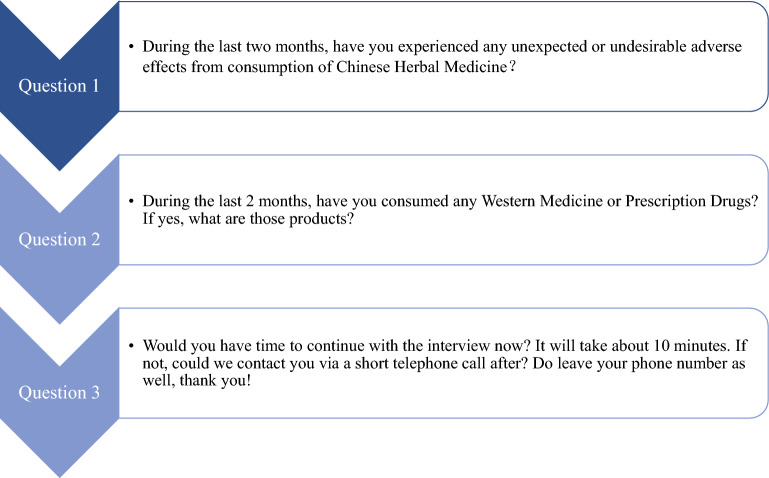
Flow of preliminary questions for patient screening

For our study, clinic patients were asked to recall their medication usage history and any AE occurrences within the past 2 months. The participating clinic staff and/or physicians asked patients three questions as listed in the Preliminary Questionnaire. The questions are shown in Fig. 2 below. If the patients answered “Yes” to Question 1 and/or Question 2, the patients would be asked to participate in a second interview using the SONAR Interview Questions. The participating clinic staff and/or physicians did not assess the causality of any reported AEs at this stage as causality assessment would only be conducted by an expert committee in Phase 2 of the study.

### Phase 2

For patients who have reported an AE from concurrent CHM and WMPD consumption and completed the SONAR Interview Questionnaire, the cases were summarized and adjudicated by a three-member committee of medical experts. The committee consisted of an expert in epidemiology, an expert in TCM herb pharmacology and an expert in Western Medicine.

Our first expert is an experienced researcher in the field, who is a senior lecturer and practicing TCM consultant at NTU TCM Clinic with over 30 years of clinical practice and is actively involved in various basic science research projects and clinical trials involving TCM herbs. She is also a member of the TCM taskforce in Singapore, and Co-Chair of TCM Research Grant Committee of Singapore’s Ministry of Health. Our second expert is trained in TCM herb pharmacology, and an experienced researcher in the field actively engaged in basic science and translational research on the prevention and treatment of human gastrointestinal cancers using CHM. Our last expert is a private general practitioner and emergency doctor with special interests in integrative Chinese and Western Medicine. He has around 10 years of experience, including working in the field of toxicovigilance in Hong Kong’s Department of Health and has published high quality research articles in internationally peer-reviewed journals. In addition, our team also had two advisors, Dr Sunita Vohra and Dr Heather Boon who conducted pharmacy SONAR in Canada, to assist and guide us in the causality assessment process. Dr Sunita Vohra is a clinician scientist with training in pediatrics, clinical pharmacology, and clinical epidemiology and her primary research interest is enhancing clinical research methods, including: (i) innovative clinical trial design; (ii) active surveillance in safety research; and (iii) improved outcomes reporting. Dr Heather Boon is the current Vice-Provost, Faculty & Academic Life of the University of Toronto and her research focuses on the safety and efficacy of traditional, complementary, and integrative health practices and products, and related regulatory and policy issues, which has been supported by over $10 million of competitive research grants.

During the causality assessment process, patient identifier information was blinded to the members of the expert committee. The adjudication process involved the presentation of the cases by the primary researcher, followed by a sharing of views and opinions from each of the three experts, after which the expert committee produced a joint assessment using both the WHO Causality Algorithm and the Naranjo Causality Scale [29, 30]. In each instance, consensus was reached through discussion.

### Statistical analysis

The data collected from Phase 1 was used to calculate population demographics by clinic. The data were first compiled into a Microsoft Excel file, before being exported for further analyses and data visualization. To evaluate any differences between the male and female groups, a two-sample *t*-test comparison was performed using IBM SPSS Statistics [31]. Thereafter, a population pyramid and box and whisker plot by gender were visualized with R Studio using the packages “tidyverse” and “ggplot2” [32, 33]. In addition to descriptive statistics, data measures calculated included standard deviation (SD), standard error mean (SEM), and 95% confidence interval (CI). The odds ratio (OR) was also calculated using IBM SPSS Statistics to determine AE occurrence likelihoods for patients who consumed both CHM and WMPD compared to CHM consumption alone. A P-value less than 0.05 was considered statistically significant.

## Results

### Baseline characteristics

A total of five outpatient clinics participated in the study over 9 weeks, lasting from 8th May 2023 to 8th July 2023. 1028 patients were screened in total. Most of the patients screened were from STCMI, accounting for 55.45% of the study population. A detailed distribution of patients by clinic is shown in Table 1 below. Additionally, it was found that most of the patients screened were of Chinese ethnicity (96.89%). In terms of education, the majority had received tertiary education (53.50%). Internal medicine (51.46%) was the main purpose of visit amongst the patients. A detailed breakdown of patient characteristics is shown in Table 2 below.

**Table 1:** Distribution of patients by clinic

Clinic	Gender	20–24	25–29	30–34	35–39	40–44	45–49	50–54	55–59	60–64	65–69	70–74	75–79	80–84	85–89	Total
*STCMI*	Male	4	6	8	5	12	12	17	19	34	34	47	31	30	2	261
	Female	7	3	6	8	10	15	26	29	45	49	41	42	26	2	309
*NTU TCM*	Male	23	10	18	6	7	5	9	7	8	10	1	5	–	–	109
	Female	11	14	19	20	3	10	9	19	7	14	–	11	1	4	142
*SCHMI*	Male	–	–	3	–	1	–	–	6	3	–	16	8	5	–	42
	Female	–	–	–	–	7	–	6	4	3	10	7	9	1	4	51
*CHHC (BWC)*	Male	–	3	4	2	8	3	2	–	2	–	–	–	–	1	25
	Female	6	9	5	14	5	12	5	3	–	–	2	–	1	–	62
*CHHC (CQC)*	Male	–	–	4	–	–	–	–	–	1	–	–	1	–	–	6
	Female	–	–	1	10	4	–	3	1	–	–	2	–	–	–	21
Total		51	45	68	65	57	57	77	88	103	117	116	107	64	13	1028

**Table 2:** Characteristics of patients screened

Characteristic	Demographic data
No. of patients screened, N (% of total)	1028 (100%)
Mean age, years	56.59 (95% CI 55.51 to 57.67)
Gender, N (% of total)	
Male	443 (43.09%)
Female	585 (56.91%)
Ethnicity, N (% of total)	
Chinese	996 (96.89%)
Malay	16 (1.56%)
Indian	4 (0.39%)
Eurasian	4 (0.39%)
Others	8 (0.77%)
Level of education, N (% of total)	
Primary	131 (12.74%)
Secondary	345 (33.56%)
Tertiary	550 (53.50%)
Others	2 (0.20%)
Purpose of visit to TCM Clinic, N (% of total)	
Internal Medicine	529 (51.46%)
Musculoskeletal	228 (22.18%)
External Medicine	74 (7.20%)
Oncology	67 (6.52%)
Fertility	64 (6.22%)
Renal	62 (6.03%)
Do not wish to disclose	4 (0.39%)

**Fig. 3:**
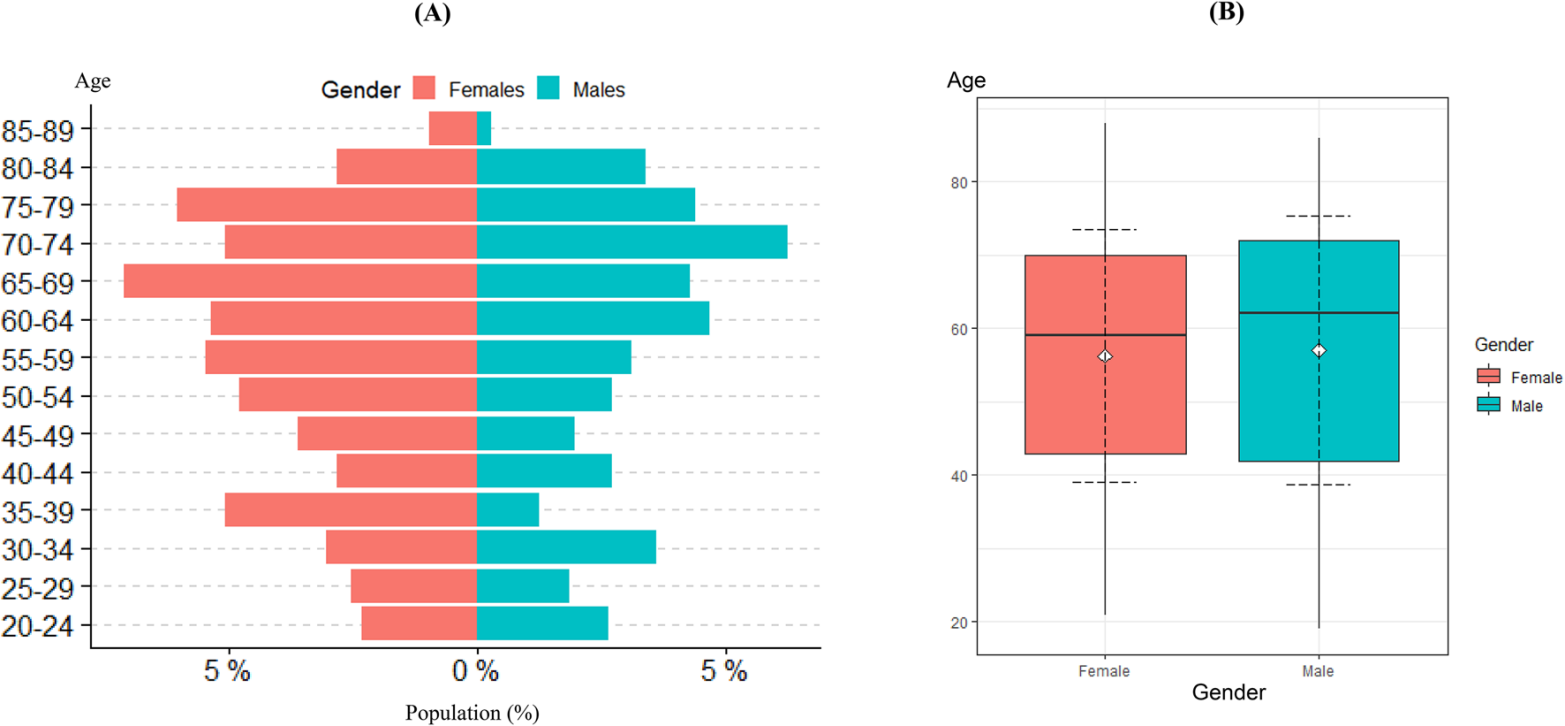
**A** Population pyramid showing demographic distribution of the participants; **B** Box and whisker plots of patients screened

In terms of gender distribution, the percentage of male and female patients were 43.09% and 56.91% respectively. The two-sample *t*-test also produced the values of *t*(1026) = 0.643 and P = 0.52, thus showing that the mean age of the male patients screened (Patients: 443, Mean: 57.00, SD:18.31; SEM: 0.87) was not significantly different from the mean age of female patients screened (Patients: 585, Mean: 56.28, SD: 17.25; SEM: 0.71). The majority of the male patient population were of the 70–74 age group, accounting for 6.23% of the overall patient population, while the majority of the female patient population were of the 65–69 age group, accounting for 7.10% of the total patient population. A population pyramid and box and whisker plot depicting the patient gender demographic distribution is depicted in Fig. 3 below.

### Phase 1: Active surveillance

From the results, it was observed that concurrent CHM and WMPD consumption was common, occurring in 62.65% (95% CI 59.69 to 65.60) of the total patients screened. Table 3 shows the proportions of participants screened who were consuming CHM, or CHM and WMPD in each clinic.

**Table 3:** Proportions of patients consuming CHM, or CHM and WMPD, by clinic

Clinic	Participants	CHM consumption only	CHM and WMPD consumption
*STCMI*	570	171 (30.00%)	399 (70.00%)
*NTU TCM*	251	138 (54.98%)	113 (45.02%)
*SCHMI*	93	27 (29.03%)	66 (70.97%)
*CHHC BWC*	87	44 (50.57%)	43 (49.43%)
*CHHC CQC*	27	4 (14.81%)	23 (85.19%)
Total	1028	384 (37.35%) 95% CI 34.40 to 40.31	644 (62.65%) 95% CI 59.69 to 65.60

Subsequently, the OR was calculated, and it was shown that patients who consumed both CHM and WMPD were 3.65 times (95% CI 1.07 to 12.48; P = 0.0389) more likely to experience an AE when compared to CHM intake alone. STCMI patients had an OR of 3.48 (95% CI 0.43 to 28.03; P = 0.242), while NTU TCM patients had an OR of 5.03 (95% CI 0.55 to 45.63; P = 0.151). For three clinics (SCHMI, CHHC BWC, CHHC CQC), an OR could not be calculated as there were no AE reports in either the CHM consumption and/or the CHM and WMPD consumption group. Table 4 shows the proportions of patients reporting AEs and the respective OR when CHM and WMPD consumption was compared with CHM consumption only.

**Table 4:** Proportions of patients reporting AEs upon consuming CHM, or CHM and WMPD, by clinic

Clinic	Participants	CHM consumption only		CHM and WMPD consumption		CHM and WMPD consumption compared with CHM consumption only	
		No AE	Reported AE	No AE	Reported AE	Odds ratio (95% CI)	P-value
*STCMI*	570	170	1	391	8	3.48 (0.43 to 28.03)	0.242
*NTU TCM*	251	137	1	109	4	5.03 (0.55 to 45.63)	0.151
*SCHMI*	93	27	–	60	6	NA	–
*CHHC BWC*	87	43	1	43	–	NA	–
*CHHC CQC*	27	4	–	23	–	NA	–
**Total**	1028	381	3	626	18	3.65 (1.07 to 12.48)	0.0389 *

Of the patients screened, 18 patients (1.75%) reported an AE after consuming CHM and WMPD while 3 patients (0.29%) reported an AE after consuming only CHM. Amongst the AE reports, the 18 patients who reported an AE from CHM and WMPD consumption were then questioned in depth using the SONAR Interview Questions to obtain further information for Phase 2 of the study. The 3 patients who reported an AE from consuming only CHM were not interviewed as our study wished to focus on potential CHM-WMPD interactions only.

### Phase 2: Causality assessment

**Fig. 4:**
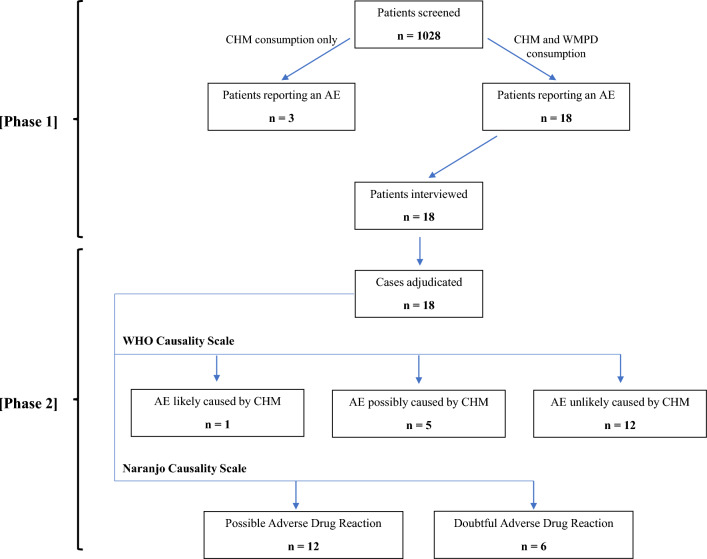
Flow chart depicting Phase 1 and Phase 2 results

A total of 18 detailed AE reports underwent causality assessment by the three-member expert committee. Following recommendations from past studies, the WHO Causality Scale and the Naranjo Causality Scale were used together for a more comprehensive evaluation [34, 35]. Firstly, adjudication with the WHO Causality Scale revealed that 1 case was likely caused by CHM, 5 cases were possibly caused by CHM, and 12 cases were unlikely to be caused by CHM. Thereafter, adjudication with the Naranjo Causality Scale revealed that 6 cases were doubtful adverse drug reactions while 12 causes were possible adverse drug reactions. A detailed flow chart depicting the results of Phase 1 and Phase 2 is shown in Fig. 4 below.

Among the AE reports adjudicated, 9 patients reported stomach discomfort, 2 patients reported fatigue and lethargy, 2 patients reported headaches, 2 patients reported nausea, 2 patients reported diarrhea, and 1 patient reported rashes and itching. A detailed summary of the cases adjudicated is provided in Table 5 below.

**Table 5:** Summary of cases adjudicated

Case	Age, Sex, Ethnicity	Smoking history, Alcohol consumption	Self-reported medication use (Oral administration unless otherwise specified)	Chinese herbal medicine consumed (Oral administration unless otherwise specified)	Adverse Event, Duration	Causality Assessment Outcome (WHO, Naranjo)
1	84, Female, Chinese	None, None	Osimertinib	*Panax ginseng, Astragalus membranaceus, Atractylodes macrocephala, Citrus reticulata, Scutellaria barbata, Scutellaria baicalensis, Hedyotis diffusai, Angelica sinensis, Radix Scrophulariae, Hordeum vulgare, Hottuynia cordata, Stemona tuberosa, Aster tataricus, Tussilago farfara,* Chicken's Gizzard-membrane*, Crataegus pinnatifida*	Persistent rash and itch, 1 year	Unlikely, Doubtful
2	34, Male, Chinese	None, None	Empagliflozin/Linagliptin	*Schisandra sinensis, Lycium barbarum, Plantago asiatica, Rubus chingii, Cuscuta chinensis, Ligustrum lucidum, Codonopsis pilosula, Atractylodes macrocephala, Poria cocos, Eucommia ulmoides, Taxillus chinensis, Epimedium grandiflorum, Anemarrhena asphodeloides, Taraxacum officinale, Citrus reticulata, Nelumbo nucifera, Lablab purpureus, Adenophora stricta, Wurfbainia villosa*	Diarrhea and feeling bloated and gassy in stomach, 3 weeks	Likely, Possible
3	56, Male, Chinese	None, None	Etoricoxib	†Herbal formula 1: Shu Jin Huo Xue Tang Additional herbs: *Morus alba, Pueraria montana, Lonicera japonica, Scutellaria baicalensis*	Malaise and lethargy, 1 week	Possible, Possible
4	62, Female, Chinese	None, None	Metformin, Aspirin	†Herbal formula 1: An Shen He Ji †Herbal formula 2: Tong Luo Hua Tan He Ji †Herbal formula 3: Bao He He Ji †Herbal formula 4: Qing Gan Ming Mu Pian	Headache and dizziness, 1 week	Possible, Possible
5	34, Male, Chinese	None, None	Hydroxyzine	*Bupleurum chinense, Paeonia lactiflora, Nepeta cataria, Saposhnikovia divaricata, Citrus reticulata, Poria cocos, Polygonum multiflorum, Dictamnus dasycarpus, Ligusticum striatum, Platycodon grandiflorus, Glycyrrhiza glabra, Paeonia suffruticosa, Gardenia jasminoides, Lycopus lucid, Radix Scrophulariae, Rehmannia glutinosa, Angelica sinensis, Ziziphus spinosa, Ossis Mastodi Fossilia*	Stomach discomfort (churning) and diarrhea, 1 month	Unlikely, Possible
6	61, Male, Chinese	None, None	Aspirin, Simvastatin	†Herbal formula 1: Shao Yao Gan Cao He Ji †Herbal formula 2: Mu Xiang Shun Qi He Ji †Herbal formula 3: Guan Jie Gu Tong Pian	Stomach discomfort (mild pain) and diarrhea, 1 week	Unlikely, Doubtful
7	76, Female, Chinese	None, None	Aspirin, Carbimazole	†Herbal formula 1: Dan Zhi Xiao Yao He Ji †Herbal formula 2: Gan Lu Yin †Herbal formula 3: Ban Xia Xie Xin He Ji †Herbal formula 4: Yue Ju Bao He Pian	Stomach discomfort (mild pain) and headache, 2 weeks	Unlikely, Doubtful
8	47, Female, Chinese	None, None	Bisoprolol, Atorvastatin, Letrozole, Trastuzumab (Intravenous infusion)	*Astragalus membranaceus, Atractylodes macrocephala, Poria cocos, Codonopsis pilosula, Glycyrrhiza glabra, Akebia quinata, Spatholobus suberectus, Coix lacryma-jobi, Ophiopogon japonicus, Schisandra sinensis, Alisma orientale, Gastrodia elata, Cibotium barometz, Eucommia ulmoides, Maydis seigma, Coptis chinensis*	Occasional nausea and vomit, 3 weeks	Unlikely, Possible
9	72, Male, Chinese	None, None	Amlodipine, Labetalol	†Herbal formula 1: Xiang Sha Liu Jun Zi Ke Li Additional herbs: *Maydis seigma, Magnolia liliiflora, Xanthium strumarium, Coptis chinensis, Forsythia suspensa, Atractylodes lancea, Alisma orientale, Citrus reticulata, Peucedanum praeruptorum, Perilla frutescens, Sinapis alba, Raphanus sativus, Descurainia sophia,* Bile Arisaema, *Lysimachia nummularia, Siegesbeckia orientalis, Agrimonia pilosa, Platycladus orientalis, Astragalus membranaceus, Spatholobus suberectus, Anemarrhena asphodeloides*	Recurring headache every morning, 2 weeks	Unlikely, Doubtful
10	31, Female, Indonesian	None, None	Estradiol, Norgestrel	*Pseudostellaria heterophylla, Schisandra sinensis, Lycium babarum, Cuscuta chinensis, Rubus chingii, Plantago asiatica, Dipsacus asperoides, Ligustrum lucidum, Angelica sinensis, Curcuma aromatica, Pinellia ternata, Scutellaria baicalensis, Prunella vulgaris, Codonopsis pilosula, Dioscorea opposita, Bupleurum chinense*	Stomach discomfort (mild pain), 1 week	Possible, Possible
11	77, Female, Chinese	None, None	Aspirin	*Paeonia lactiflora, Bupleurum chinense, Poncirus trifoliata, Magnolia officinalis, Angelica sinensis, Pinellia ternata, Perilla frutescens, Prunus armeniaca, Raphanus sativus,* Chicken's Gizzard-membrane, Medicated Leaven, *Trichosanthis Radix, Polygonum multiflorum, Platycladus orientalis, Ziziphus spinosa, Ligusticum striatum, Astragalus membranaceus, Pseudostellaria heterophylla, Aloe vera, Glycyrrhiza glabra*	Stomach-ache (cramping sensation), 1 week	Possible, Possible
12	32, Male, Chinese	None, None	Codeine, Fexofenadine, Estazolam	*Bupleurum chinense, Angelica sinensis, Poria cocos, Atractylodes macrocephala, Paeonia lactiflora, Glycyrrhiza glabra, Paeonia suffruticosa, Gardenia jasminoides, Mentha canadensis, Ziziphus spinosa, Lilii bulbus, Codonopsis pilosula, Ophiopogon japonicus, Schisandra sinensis, Ossis Mastodi Fossilia, Ostrea edulis*	Fatigue and sleepiness, 1.5 weeks	Unlikely, Possible
13	42, Female, Chinese	None, None	Propanolol	†Herbal formula 1: Liu Wei Di Huang Wan †Herbal formula 2: Dan Zhi Xiao Yao San Additional herbs: *Eucommia ulmoides, Radix Scrophulariae, Prunella vulgaris, Ostrea edulis, Fritillaria thunbergii, Anemarrhena asphodeloides, Phellodendron amurense, Pinellia ternata, Citrus reticulata, Forsythia suspensa, Pueraria montana, Coix lacryma-jobi*	Stomach discomfort (churning) and bloating, 1 week	Unlikely, Possible
14	74, Female, Chinese	None, None	Rivaroxaban, Rabeprazole	*Astragalus membranaceus, Spatholobus suberectus, Codonopsis pilosula, Atractylodes macrocephala, Pogostemon cablin, Pueraria montana, Ophiopogon japonicus, Citrus reticulata, Cimicifuga simplex, Bupleurum chinense, Paeonia lactiflora, Poncirus trifoliata, Saposhnikovia divaricata, Heracleum hemsleyanum, Cibotium barometzou, Moghania philippinensis, Achyranthes bidentata, Coix lacryma-jobi, Nelumbo nucifera, Glycyrrhiza glabra*	Recurring diarrhea after food or medicine consumption, 1 week	Unlikely, Possible
15	53, Female, Chinese	None, None	Prospan, Acetylcysteine	†Herbal formula 1: Chuan Xiong Cha Tiao San †Herbal formula 2: Xin Yi Tang †Herbal formula 3: Zhe Bei He Ji †Herbal formula 4: Ming Mu Di Huang Wan †Herbal formula 5: Yi Qi Sheng Jin Jiao Nang	Recurring diarrhea 10 min after CHM consumption, 4 days	Possible, Possible
16	23, Male, Chinese	None, None	Acetaminophen, Dequalinium lozenges	†Herbal formula 1: Sha Shen Mai Dong Tang Additional herbs: *Taraxacum officinale, Dendrobium officinale, Trichosanthis Radix, Coix lacryma-jobi, Dioscorea opposita, Rehmannia glutinosa, Cyperus rotundus, Bupleurum chinense*	Occasional nausea after CHM or WMPD consumption, 2 weeks	Unlikely, Doubtful
17	53, Female, Chinese	None, None	Pembrolizumab(Intravenous infusion)	†Herbal formula 1: Yang Yin Qing Fei Ke Li Additional herbs: *Spatholobus suberectus, Liquidambar formosana, Akebia quinata, Astragalus membranaceus, Ziziphus spinosa, Polygala sibirica, Dendrobium officinale, Lilii bulbus*	Recurring dull stomach pain, 1 week	Unlikely, Doubtful
18	84, Male, Chinese	None, None	Aspirin, Tamsulosin, Carbimazole	*Rosa laevigata, Mantidis ootheca, Lindera aggregata, Alpinia oxyphylla, Glycyrrhiza glabra, Schisandra sinensis, Panax ginseng, Astragalus membranaceus, Cinnamomum verum, Rehmannia glutinosa,* Donkey-hide Gelatin, *Ophiopogon japonicus, Ziziphus jujuba, Spatholobus suberectus, Liquidambar formosana, Achyranthes bidentata, Heracleum hemsleyanum*	Stomach discomfort (churning) and diarrhea, 3 days	Unlikely, Possible

^†^Products and ingredients are as stated on the manufacturers’ product package

## Discussion

### Main findings

The implementation of active surveillance in outpatient TCM clinics allowed for the detection of AEs reported by patients consuming CHM and/without WMPD. A total of 1028 individuals were screened for our study, and it was discovered that concurrent CHM and WMPD intake was prevalent, occurring in 62.65% (95% CI 59.69 to 65.60) of the total patients examined. Clinic patients who consumed both CHM and WMPD were 3.65 times (95% CI 1.07 to 12.48; P = 0.0389) more likely to experience an AE than those who used CHM alone.

In addition, our study’s interview and adjudication process allowed for complete causality assessment of the reported AEs. The participation of an expert committee comprising of professionals in epidemiology, TCM herb pharmacology, and Western Medicine allowed for a thorough evaluation and exchange of meaningful insights from multiple perspectives, thus enhancing the comprehensiveness of the adjudication process. Amongst the cases adjudicated, most patients reported that their reported AEs did not interfere with their ability to conduct regular daily activities. Based on the evaluations from the two causality scales, we found that AE occurrence is likely to be multi-factorial and not solely attributed to the consumption of both CHM and WMPD. Although we could not identify the exact causes just by the two causality scales, we could further conduct more animal studies or toxicity studies to investigate the underlying mechanisms of action.

Lastly, our study found that the incidence of patient-reported AEs in TCM clinic settings was low, with only 21 patients (2.04%) reporting either an AE arising from solely CHM or from CHM and WMPD consumption. In contrast, the level of severity and AE occurrences in other healthcare practices were found to be greater. For instance, a recent scoping review of 25 studies investigating in-hospital AEs revealed that approximately 10% of hospital patients reported at least one AE, with 7.3% of these AEs being fatal [36]. A similar study on the prevalence of AEs in home healthcare populations in America also discovered that 13% of home healthcare patients reported an AE, with more than 75% of reported AEs being post-discharge related and requiring prolonged patient support [37]. Therefore, the low AE rate and mild nature of reported AEs in our study is a positive indication, and local TCM institutions should strive to further improve patient safety. Lastly, as compared to the pharmacy SONAR study in Canada, which also used a similar outpatient study model, their study indicated that 7.3% of study participants reported an AE from concurrent use of natural health products and prescription medications.

### Strengths

One strength of our study was the large sample size of 1028 patients across five TCM outpatient clinics. To date, this is the first and largest TCM related multi-center observational study on CHM-WMPD adverse reaction conducted in Singapore. The inclusion of commercial, nonprofit, and educational institutions provided a good overview and sampling of the nation’s TCM patient population, allowing us to gain a better understanding of patient demographics and medicine consumption patterns.

Furthermore, post-study feedback from the participating clinics revealed that the preliminary screening questions were quick and straightforward, and were well accepted by patients, participating clinic personnel, and physicians. The use of brief and succinct screening questions allowed for swift and efficient screening while also causing little dissatisfaction to the patients. The physician was also made aware of the patient's existing medication consumption history through this screening method, allowing them to better formulate prescriptions to complement the WMPD consumed by the patients, if any. Hence, the implementation of active surveillance into TCM AE collection is one key strength of our study and hopefully, this would help further enhance the comprehensiveness of TCM clinical diagnosis and treatment.

## Limitations

### Potential sources of bias

The first potential source of bias identified was the possibility of sampling bias, as not all patients were screened due to the hectic clinic schedule. Due to the physicians and clinic staff’s busy schedules, the screening of patients depended largely on their workload. Furthermore, as this project had no form of monetary funding and incentive, all external assistance was voluntary, therefore physicians and clinic personnel may not have prioritized the screening and data collection as it was an imposition on their hectic schedules as well. As a result, the primary researcher was responsible for most of the screening and data collecting, rather than the physicians and clinic personnel. Considering this limitation, the following strategies were applied to counter these challenges. Firstly, recently graduated physicians with prior research experience were more actively engaged to assist in the patient screening process. This was advantageous as they were more likely to actively participate in the study process. Secondly, numerous site visits were made to ensure that the research protocol was adhered to. The primary researcher visited each study site once a week to conduct patient screening and to ensure seamless study implementation throughout the whole study period.

Another source of bias identified could have been recall or response bias. It was possible that the patient was not able to provide accurate information to the interviewer, thus leading to possible inaccuracies in data collection. To mitigate this constraint, the primary researcher verified the information supplied by respondents with the attending physician in as many cases as possible. In addition, the information supplied by the patients for the AE reports was cross-checked against patient records in the clinic database to confirm that the information provided was accurate. The patient’s medication records were also verified against their “Health Hub” application, which stores the patient’s prescription medication and health check records. This was especially useful in acquiring the patients' specific CHM and WMPD prescriptions, as they may not be able to recollect all details properly during the interview procedure.

### Variations in results

One key variation identified through our study was the uneven ethnic composition of our patient population as compared to the actual ethnic composition in Singapore. In our study, Chinese patients formed the vast majority, accounting for 96.89% of the total patient population. This might be explained by TCM's profound roots in Chinese culture, which may be more appealing to the Chinese population due to its long-standing history [41]. Although this trend may be within expectations, it could affect the generalizability of study findings to different patient ethnic groups. Future epidemiological research might take this factor into account during the planning of the study design and strive to maintain a well-balanced ethnic diversity to improve the generalizability of study findings.

Another key variation identified was the difference in AE rates across the different TCM outpatient clinics. The lack of AE reports in either the CHM consumption or the CHM and WMPD consumption group in some clinics did not allow for the calculation of their respective AEs. One probable explanation is that the overall number of patients at these clinics was insufficient to capture an accurate AE rate. As privatized clinics, CHHC BWC and CHHC CQC screened fewer people than SCTMI and SCHMI, which were nonprofit institutions that offered significantly subsidized TCM consultations, thus attracting a higher patient flow. Therefore, more research is required to evaluate the validity of these reported variances.

Lastly, variation was observed when causality assessment was undertaken using two different assessment scales. Using the WHO Causality Scale, 1 case was determined to be likely caused by CHM, 5 cases possibly caused by CHM, and 12 cases unlikely caused by CHM. On the other hand, 6 cases were determined to be doubtful adverse drug reactions while 12 cases were determined to be possible adverse drug reactions using the Naranjo Causality Scale. A literature search also revealed that this trend was detected in similar investigations. For instance, in a study conducted between 2016 and 2018 at the Department of Pharmacology at the All India Institute of Medical Sciences Bhopal, two independent groups analyzed 842 AE case reports using either the WHO or the Naranjo Causality Scale [30]. However, the Cohen's kappa coefficient (κ) statistical test, demonstrated that there was no agreement between the WHO and Naranjo Causality Scales. Studies conducted by other research groups also yielded similar results, with the kappa coefficient statistical test demonstrating low agreement between the WHO and Naranjo Causality Scales [34, 42, 43]. Given these findings, adapting an active surveillance model to detect the AEs from herb-drug interactions is essential to improve the quality and reliability of further causality assessments.

### Possible explanations and further studies

Amongst the clinics involved in the study, the common practice was to advise patients to space out the consumption of CHM and WMPD by 2 h to minimize possible incidences of herb-drug interactions [44]. This is a common clinical practice enforced in Singapore TCM clinics, and is often provided in the physician’s instructions to patients to consume CHM 2 h apart from WMPD [45, 46]. However, one possible limitation lies in the different half-lives of different drugs. For example, diabetic drugs such as metformin and linagliptin have lengthy half-lives in plasma (2.7 h for metformin and 12 h for linagliptin) as compared to blood thinning agents like aspirin which has a shorter half-life of 20 min [47–49]. With variances in drug half-lives, it is thus difficult to predict the rates of drug clearance from our body systems. Furthermore, hepatic and renal diseases may interfere with the body's metabolism and elimination of the drugs, resulting in the drugs being present in the patient's body systems for an extended period of time [50, 51]. Hence, the recommended time gap of 2 h might not be sufficient to account for the plasma clearance of the WMPD. Therefore, based on the patient's prior diseases and medication use, it is essential that physicians reconsider the acceptable time gap between CHM and WMPD consumption and counsel patients appropriately to ensure patient safety.

Another critical point to emphasize is herbal bioactivation. While the molecular processes behind herbal compound-related AEs are not well elucidated, recent research has discovered that bioactivation of herbal compounds could potentially generate reactive intermediates [52, 53]. Like drugs, constituents of herbal compounds can also undergo Phase I and Phase II reactions to form nontoxic metabolites, which are eventually excreted from the body. However, reactive and potentially toxic metabolites could also be formed in the process, which could be an underlying cause of AE occurrences [54]. Some examples of known herbal compounds undergoing bioactivation associated with clinical toxicity include aristolochic acid and furans [55, 56]. In addition, the use of CHM may cause synergistic or antagonistic interactions with concomitant drugs and/or modify drug disposition, resulting in therapeutic failure or toxicity [57]. Moving forward, research could thus focus on the following areas. Firstly, reliable toxicity biomarkers should be utilized to identify and predict the occurrence of AEs. Furthermore, basic research should try to better understand the mechanisms of action behind herbal bioactivation. Together with existing knowledge on the pharmacokinetics and pharmacodynamics of WMPD, this would allow for a better understanding of both the fates of drugs and herbs upon consumption, as well as ensuring that CHM and WMPD exert their effects in a complementary manner rather than contributing to AE occurrences.

In addition, a common observation in clinical practice was physicians prescribing multiple herbal formulas together in treatment. This is due to TCM’s belief that multiple ingredients in herbal formulas targeting multiple targets could produce synergistic or cumulative effects, thus enhancing therapeutic potential [58]. Novel techniques such as network pharmacology have also attempted to elucidate the synergistic effect of TCM compounds [59, 60]. One such herb highlighted by one of the pharmacological experts during causality assessment was *Glycyrrhiza glabra. Glycyrrhiza glabra* is known as a “courier medicinal” herb which serves to regulate the other herbs in the same formula [61]. As a result, *Glycyrrhiza glabra* is prevalent in TCM formulations, and studies have indicated that it is a primary component in almost 60% of all TCM prescriptions [62]. Although *Glycyrrhiza glabra* possesses anti-inflammatory, immunostimulatory, and antiviral bioactivities, it has been demonstrated that excessive and prolonged usage of *Glycyrrhiza glabra* may result in pseudoaldosteronism [63]. Glycyrrhetinic acid, a key component of *Glycyrrhiza glabra*, has been shown to (1) block the 11-hydroxysteroid dehydrogenase 2 enzyme, which is responsible for the breakdown of active cortisol into inactive cortisol, (2) and bind to the mineralocorticoid receptor as an agonist [64, 65]. As a result of high glycyrrhetinic acid levels, cortisol is unable to be deactivated and instead binds and activates the mineralocorticoid receptor, resulting in pseudoaldosteronism. Common presenting symptoms include headaches, weariness, and high blood pressure [66]. Hence, TCM practitioners must be aware of the recommended safe dosages of herbs, particularly when there are repeating herbs in their prescribed formulas.

### Future directions

#### Improving clinical practice

Active surveillance was found to be applicable in detecting AEs in this trial. One clinical application may be as follows. Doctors should try to include these screening questions in their diagnostic approach to better understand their patients' current medication consumption history and to know which herbs to avoid in prescriptions. By increasing the rates of AE identification and reporting, possible risk factors may be identified early, potentially preventing the transition from an AE to an AR. The preliminary screening questions were also brief and simple, lasting only around 30 s for each patient. Therefore, this should lower the number of AEs in the clinical environment, thus increasing overall patient safety.

At this present moment, an Adverse Event Online Database exists in Singapore and can be found on the Health Sciences Authority website [67]. In Singapore, there is a sizable, patented medicine industry for both acute and chronic disorders [68]. Hence, the Health Sciences Authority has formed a Product Vigilance Advisory Committee comprised of professionals in medicine, pharmacy, pharmacology, and forensic sciences responsible for analyzing important drug safety concerns and advising on the relevant regulatory steps to improve drug safety [69]. However, TCM practitioners do not have access to this database, and it is only accessible to the WM practitioners. This might be understandable, given that the primary focus of these surveillance methods is focused on WM products such as vaccines and new drugs undergoing clinical trials [70–72]. Nonetheless, it would be advantageous for authorities to offer both TCM and WM practitioners joint access to this database to monitor AEs from both TCM and WM patients. This would also allow TCM practitioners to assist with AE reporting and monitoring, thus contributing to improved patient safety.

Additionally, data acquired in this study could potentially be included into a database that TCM and WM doctors could access. With a database of combinations used with and without AEs, TCM and WM professionals might be better equipped to know which combinations to avoid when administering medicine. According to a recent summary by Zhang et al. in 2022, some examples of freely accessible databases include The Chinese-Western Medicine Integrative Information Network and Probot Chinese Medicine-Drug Interaction Database; while examples of commercially available databases include the UW Drug Interaction Database and Natural Medicines Comprehensive Database [73]. Hence, a similar model could be applied in Singapore to include updated herb-drug interactions, which could be implemented and shared across major healthcare providers nationwide such as clinics and hospitals. Together with the input from TCM and WM healthcare professionals, the creation of such a database should aim to be constantly revised and supplemented to present healthcare professionals with comprehensive and correct information to aid with medicine prescription. In the future, this could potentially be beneficial to improving overall patient safety.

### Future research directions

As shown in this study, we have found that the rates of concomitant usage of CHM and WM were high in the Singapore environment. As a result, there may be more instances of possible herb-drug interactions. Therefore, future research might concentrate on better understanding the molecular mechanisms of actions of possible herb-drug interactions. The data collected through preliminary screenings could be used in collaborations with pharmacology professionals and researchers to identify potential compounds that led to reported AEs, and these compounds could be sent for chemical and pharmacological analysis to elucidate possible mechanisms of herb-drug interactions.

Additionally, future research should also concentrate on certain patient groups with a greater rate of reported AEs, such as those with oncological or renal disorders. Concurrent use of CHM and WM was highly prevalent (100% for renal patients, 82.10% for oncology patients), thus putting these patient groups at a greater risk of possible herb-drug interactions [74, 75]. In addition, the drugs prescribed for these disorders, such as anti-diabetic or anti-cancer drugs tend to have longer half-lives to maintain a prolonged effect. This also contributes to an increased risk of potential herb-drug interactions. Thus, future studies could focus on these high-risk patient groups to better understand the CHM and WMPD consumption patterns, and respective AE rates of these patients. Subsequent studies could then attempt to investigate the respective pharmacokinetic and pharmacodynamic properties of these drugs together with that of prescribed CHM to avoid their concurrent presence in plasma before complete clearance of the other. Hopefully, these initiatives will contribute to improved patient safety in the long run. In addition, our results show that AE occurrence likelihood for patients who consumed both CHM and WMPD is higher compared to CHM consumption alone. However, it remains unclear what the likelihood is of CHM and WMPD consumption as compared to WMPD consumption alone. Hence, future studies should also consider the rate of AE among patients using WMPD alone. It would also be beneficial to employ qualitative methods such as patient interviews or focus groups to gain a deeper understanding of patients' perspectives and experiences. This would allow research groups to then better understand the experience of AE related to the use of CHM.

Lastly, our study found that concurrent administrations of CHM and WMPD increased risks of AEs occurrence as compared to CHM treatment alone. Hence, there could be a possibility that concurrent administrations of CHM and WMPD could reduce toxicity of WMPD. In TCM, preparation procedures such as the processing of the raw materials, decoction, and drug compatibility to the identification of the patient’s constitution and adjustment of remedies, aim to enhance the therapeutic efficacy and reduce toxicity at the same time [76]. The preparation of TCM using various different adjuvants, such as vinegar, wine, or honey, contributes significantly to the change in chemical profile and pharmacological effects and toxicity of TCM drugs [77]. When used together, it has also beenshown that CHM could potentially lower the toxicity of some WMPD. For instance, *Forsythia* and *Ginger* were used to reduce nausea and vomiting caused by the treatment of digoxin [78]. *Poria* and *Eucommia* were also added in decoction formulas to prevent the occurrence of hypokalemia and hyponatremia resulting from diuretic consumption [79]. Additionally, an integrative approach of concurrent Chinese and Western medicine consumption has also been shown to reduce chemo-toxicity in cancer therapy while maintaining or even enhancing therapeutic effects [80]. However, as our study did not involve basic research, we could not validate possible herb-drug interactions from our case studies on a biomolecular level at this point. Hence, future studies could consider the inclusion of laboratory analysis together with causality assessment to allow for better validation of herb-drug interactions.

**Fig. 5:**
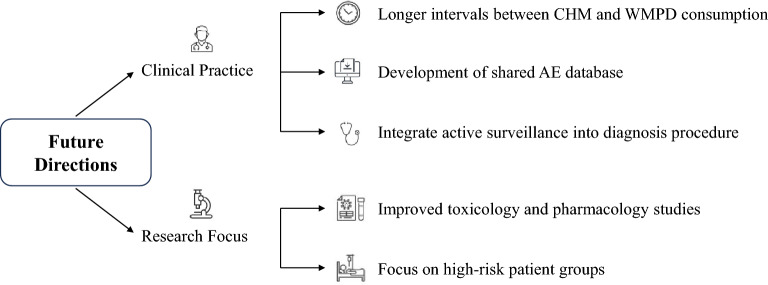
Summary diagram of implications on clinical practice and future research directions

A summary diagram detailing the proposed implications on clinical practice and future research directions is shown in Fig. 5 below.

## Conclusion

In closing, our study has shown that implementation of active surveillance for the detection of CHM-WMPD related AEs in local TCM clinics is feasible. We also found that concurrent CHM-WMPD consumption was prevalent and patients who consumed CHM and WMPD were 3.65 times more likely to experience an AE as compared to CHM consumption alone. The AEs reported were all milder in nature and most AEs were deemed unlikely due to CHM consumption.

In addition, feedback received from the participating clinics has also been positive, thus supporting implementation into the diagnosis protocols of TCM physicians. Nonetheless, future studies might concentrate on the following areas. In terms of clinical practice, we propose extending the time between CHM and WMPD consumption, creating a common AE database, and incorporating active screening into TCM diagnostic processes. In terms of research focus, we recommend a greater emphasis on the toxicity and pharmacology of CHM and WMPD interactions, as well as a greater emphasis on herb-drug interactions in high-risk patient groups.

## Data Availability

All data sources described in this research are available from the corresponding authors.

## Abbreviations

AE: Adverse event
AR: Adverse reaction
CAM: Complementary and alternative medicine
CHHC BWC: Chong Hoe Healthcare Beauty World Centre Branch
CHHC CQC: Chong Hoe Healthcare Clarke Quay Central Branch
CHM: Chinese Herbal medicine
CI: Confidence interval
NTU TCM: NTU Chinese Medicine Clinic
OR: Odds Ratio
SCHMI: Singapore Chung Hwa Medical Institution
SD: Standard deviation
SEM: Standard error mean
SONAR: Study Of Natural health product adverse reactions
STCMI: Singapore Thong Chai Medical Institution
TCM: Traditional Chinese Medicine
WM: Western Medicine
WMPD: Western Medicine Prescription Drugs
